# PCMT1 Is a Potential Prognostic Biomarker and Is Correlated with Immune Infiltrates in Breast Cancer

**DOI:** 10.1155/2022/4434887

**Published:** 2022-04-30

**Authors:** Jufang Guo, Xuelian Du, Chaolin Li

**Affiliations:** Department of Obstetrics and Gynecology, Jinniu District Maternal and Child Health Hospital, Chengdu, China

## Abstract

**Background:**

Protein-L-isoaspartate (D-aspartate) O-methyltransferase (*PCMT1*) is involved in the occurrence and development of a variety of malignant tumors. However, the prognostic value of *PCMT1* in breast cancer remains unclear.

**Methods:**

Based on the Cancer Genome Atlas database, we assessed the correlation between the expression of *PCMT1* and prognosis, immune invasion, and tumor mutation burden in a variety of cancers. The expression level, mutation, immune correlation, and coexpression of *PCMT1* in breast cancer were studied using the following databases: UALCAN database, Human Protein Atlas database, cBioPortal database, TIMER database, and LinkedOmics database. Kaplan–Meier Plotter was used for survival analysis. Receiver operating characteristic (ROC) curves and nomograms were drawn using the R software package. *P* < 0.05 was considered statistically significant.

**Results:**

Pancancer analysis showed that *PCMT1* is highly expressed in a variety of cancers and is significantly related to the prognosis of a variety of cancers. *PCMT1* is significantly related to the tumor mutation burden of a variety of cancers. *PCMT1* is significantly high in breast cancer, and it is significantly related to the abundance of immune infiltration. Survival analysis revealed that high *PCMT1* expression is significantly associated with shorter overall survival (OS), relapse-free survival (RFS), and postprogression survival (PPS) in breast cancer patients. ROC curves and nomograms verify the effectiveness of *PCMT1* as a prognostic biomarker for breast cancer.

**Conclusions:**

PCMT1 can be used as a potential prognostic biomarker of breast cancer, and it is significantly related to the abundance of breast cancer immune infiltration.

## 1. Introduction

Breast cancer (BRCA) is one of the most common malignant tumors in women worldwide [1]. The annual incidence and fatality rate of breast cancer ranks at the forefront of all cancers [2]. Statistics show that among the most common cancers diagnosed in American women in 2021, breast cancer alone accounts for 30% of female cancers. In 2021, there will be 284,200 new breast cancer patients in the USA, of which 44,130 deaths are estimated [3]. However, there are still many unclear parts of the specific molecular biological mechanism of breast cancer occurrence and development. In addition, the early diagnosis rate of breast cancer is low, and the side effects of treatment are large, resulting in the overall effect of clinical breast cancer treatment being unsatisfactory [4–6].

At present, the main means to reduce the incidence and mortality of breast cancer is to find specific diagnostic criteria and increase the sensitivity and specificity of early diagnosis [7, 8]. In recent years, biomarkers have attracted wide attention because of their ability to predict tumor development, prognosis, and response to treatment [9]. The emergence of molecular biomarkers of breast cancer is aimed at improving the early diagnosis rate of breast cancer, predicting the effects of related prognostic treatments, and providing better clinical guidance [10]. With the update of detection technology and the continuous development of immunotherapy, an increasing number of breast cancer biomarkers have been used clinically. However, based on the limited clinical specificity and sensitivity, the development of new breast cancer biomarkers is still very urgent [11].

Protein-L-isoaspartate (D-aspartate) O-methyltransferase (*PCMT1*) is an S-adenosylmethionine-dependent methyltransferase. The main functions of *PCMT1* are to initiate the repair of damaged proteins by catalyzing methyl esterification of L-isoaspartyl and D-aspartyl residues produced by spontaneous isomerization and racemization of L-aspartyl and L-asparaginyl residues in aging peptides and proteins. Previous studies have shown that strong *PCMT1* expression is not only a predictive marker for poor prognosis of surgically removed lung adenocarcinoma but also an unfavorable prognostic biomarker for bladder cancer [12–14]. It participates in cell migration and invasion by regulating epithelial-mesenchymal transition-related genes and acts as an oncogene in bladder cancer [15]. In addition, the latest research shows that *PCMT1* promotes the migration and invasion of human U-87 MG and U-251 MG glioblastoma cell lines and plays a key role in the growth of glioblastoma [16]. These studies show that *PCMT1* is very important for the occurrence and development of a variety of malignant tumors. Through pancancer and related bioinformatics analysis of *PCMT1* in breast cancer, it will help us understand the related functions of *PCMT1* in breast cancer and provide new insights for breast cancer drug development and clinical exploration of molecular markers for diagnosis and prognosis.

## 2. Materials and Method

### 2.1. Pancancer Analysis of PCMT1

We downloaded a unified standardized pancancer data set from the UCSC (https://xenabrowser.net/) database: TCGA pancancer (PANCAN, *N* = 10535, *G* = 60499), and then, we extracted the PCMT1 gene expression data in each sample (sample sources include solid normal tissue and primary tumor tissue). The log2 (*x* + 0.001) transformation was performed on each expression value. Finally, we also eliminated the cancer species with less than 3 samples and finally obtained the expression data of 26 cancer species [16, 17]. First, we used R software v4.0.3 to compare the expression of *PCMT1* in a variety of cancer tissues and normal tissues. The DiffExp module in the TIMER database allows users to study the differential expression of PCMT1 between tumors and adjacent normal tissues in all TCGA tumors. The *PCMT1* pancancer prognosis analysis adopts univariate Cox regression analysis and is realized by the “forestplot” R package, using forest plots to display the *P* value, hazard ratio (HR), and 95% confidence interval (CI) of each variable. To perform a reliable immune correlation assessment, we used the R software package Immunedeconv, which integrates the six latest algorithms, including TIMER, xCell, MCP-counter, CIBERSORT, EPIC, and quanTIseq [18–20]. *SIGLEC15*, *IDO1*, *CD274*, *HAVCR2*, *PDCD1*, *CTLA4*, *LAG3*, and *PDCD1LG2* are transcripts related to immune checkpoints [21]. We extracted the expression values of these 8 genes and observed the expression of immune checkpoint-related genes. Tumor mutation burden was derived from the article, the immune landscape of cancer, published by Vesteinn Thorsson et al. in 2018 [22]. The Spearman correlation analysis between tumor mutation burden and *PCMT1* gene expression was calculated using R software v 4.0.3. The rank-sum test was used to detect the two sets of data, and a *P* value of <0.05 was considered statistically significant.

### 2.2. Analysis of PCMT1 Expression in Breast Cancer

We downloaded the unified normalized TCGA-BRCA data set (tumor samples =1092; normal samples =292) from the UCSC (https://xenabrowser.net/) database. The expression data of the *PCMT1* gene in each sample were extracted, and log2 (*x* + 0.001) transformation was performed on each expression value. Expression differences between normal and tumor samples were calculated using R software (version 3.6.4), and significant differences were analyzed using unpaired Wilcoxon rank sum and signed rank tests. And finally, the expression value of *PCMT1* in normal breast and breast tumors was obtained. In addition, we downloaded the GSE3744 data set (tumor samples =40; normal samples =7) from the GEO database for validation of differential expression. The UALCAN database (http://ualcan.path.uab.edu/) can be used to analyze cancer omics data [23]. We used TCGA sample analysis to evaluate the expression of PCMT1 protein in breast cancer, the level of *PCMT1* methylation, and the correlation with tumor staging. The Human Protein Atlas (HPA) network database (https://www.proteinatlas.org/) further evaluated the protein expression of *PCMT1* in clinical breast cancer samples, which contains more than 10 million immunohistochemistry images of various human cells, tissues, and organs [24]. The Cancer Cell Line Encyclopedia (CCLE) database is an online database that can freely explore the genetic information of 947 human tumor cell lines [25]. We used CCLE to evaluate the expression level of *PCMT1* in different breast cancer cell lines. The expression level of *PCMT1* in breast cancer cell lines was converted from log2 and plotted as a heat map. Breast cancer gene-expression miner (bc-GenExMiner) v4.7 (Updated on June 28, 2021) contains a large amount of breast cancer genome data, which can perform statistical analysis on expression, prognosis, and correlation [26]. The relationship between the PCMT1 gene and the clinicopathologic parameters of breast cancer were analyzed by using bc-GenExMiner v4.7.

### 2.3. PCMT1 Mutation and Immune Infiltration in Breast Cancer

The cBioPortal for cancer genomics (https://www.cbioportal.org) is based on a multidimensional cancer genomics data set, providing fast, intuitive, and high-quality access to the molecular profile and clinical attributes of large-scale cancer genomics projects [27]. We explored *PCMT1* mutations in the clinical breast cancer data set (TCGA, Firehose Legacy, 1108 total samples). TIMER (https://cistrome.shinyapps.io/timer/) aims to systematically analyze the results of immune infiltration of multiple cancers [28]. We used TIMER to evaluate the correlation between the expression of *PCMT1* in breast cancer and the abundance of B cells, CD8+ T cells, CD4+ T cells, macrophages, neutrophils, and dendritic cells in breast cancer. The results are displayed as a scatter plot, showing the statistical significance and the purity-corrected part of Spearman's rho value. We also evaluated the correlation between high and low expression of *PCMT1* and breast cancer immune cell infiltration based on ssGSEA (single sample gene set enrichment analysis) and EPIC algorithm. TIP (tracking tumor immunophenotype) can be used to analyze seven anticancer immune states and the proportion of tumor-infiltrating immune cells. The seven anticancer immune states include release of cancer cell antigens (step 1), cancer antigen presentation (step 2), priming and activation (step 3), trafficking of immune cells to tumors (step 4), infiltration of immune cells into tumors (step 5), recognition of cancer cells by T cells (step 6), and killing of cancer cells (step 7). We calculated the correlation between high and low expression of *PCMT1* and breast cancer immune activity score based on the TIP database [29].

In addition, based on the mRNA sequence data of TCGA breast cancer, we analyzed the Spearman correlation between PCMT1 expression and tumor mutation burden (TMB) and microsatellite instability (MSI). Immune checkpoint blockade (ICB) therapy has completely changed the treatment of human cancer. Based on the expression profile data, we use the tumor immune dysfunction and exclusion (TIDE) algorithm to predict the responsiveness of PCMT1 expression to predictive immune checkpoint inhibitors [30]. A high TIDE score means that immune checkpoint blocking (ICB) has poor efficacy and short survival after ICB treatment.

### 2.4. PCMT1 Coexpressed Genes in Breast Cancer

The LinkedOmics database (http://www.linkedomics.orglogin.php) is a publicly available portal that includes multiomics data from all 32 TCGA cancer types [31]. We used this database to identify genes closely related to *PCMT1* and combined the cluego [32] and cluepedia [33] plug-ins in Cytoscape [34] and Metascape [35] to perform functional enrichment analysis of the first 50 coexpressed genes of *PCMT1*.

### 2.5. Kaplan–Meier Plotter

Based on the online server Kaplan–Meier (KM) Plotter (https://kmplot.com/analysis/) [36], we evaluated the prognostic value of *PCMT1* for overall survival (OS), relapse-free survival (RFS), distant metastasis-free survival (DMFS), and postprogression survival (PPS) in breast cancer. *P* < 0.05 is considered statistically significant.

### 2.6. The Influence of the PCMT1 Gene on the Prognosis of Breast Cancer

We downloaded the raw counts and corresponding clinical information of the RNA sequencing data (level 3) of 1,097 breast cancer tumors from the TCGA data set. The log rank was used to test the KM survival analysis to compare the survival differences between the above two or more groups, and timeROC analysis was performed to compare the prediction accuracy and risk score of *PCMT1*. Gene expression and survival time and survival status in the TCGA data set were implemented through the R software package ggrisk; the KM survival curve distribution of *PCMT1* in the TCGA data set was implemented through the R software packages survival and survminer. The receiver operator characteristic curve (ROC) and area under the curve (AUC) of *PCMT1* at different times were determined by the R software package timeROC [37]. For the KM curve, the *P* value and the HR with 95% CI were obtained by log rank test and univariate Cox proportional hazard regression. All the above analysis methods and R software packages were implemented using v4.0.3 version R software (R Foundation for Statistical Computing, 2020). *P* < 0.05 was considered statistically significant.

### 2.7. Construction and Verification of Nomogram

We assessed the impact of *PCMT1* and clinical factors such as age, sex, race, PT stage, and PN stage on prognosis. First, univariate and multivariate Cox regression analyses were performed, and forest plots were generated with the “forestplot” R package to display the *P* value, HR, and 95% CI of each variable. According to the results of multivariate Cox proportional hazard analysis, the R software package “rms” was used to establish a nomogram to predict the total recurrence rate in 3 years. The nomogram provides a graphical representation of these factors, and the prognostic risk of a single patient can be calculated through the points related to each risk factor [38].

## 3. Results

### 3.1. Pancancer Analysis Results of PCMT1

**Figure 1:**
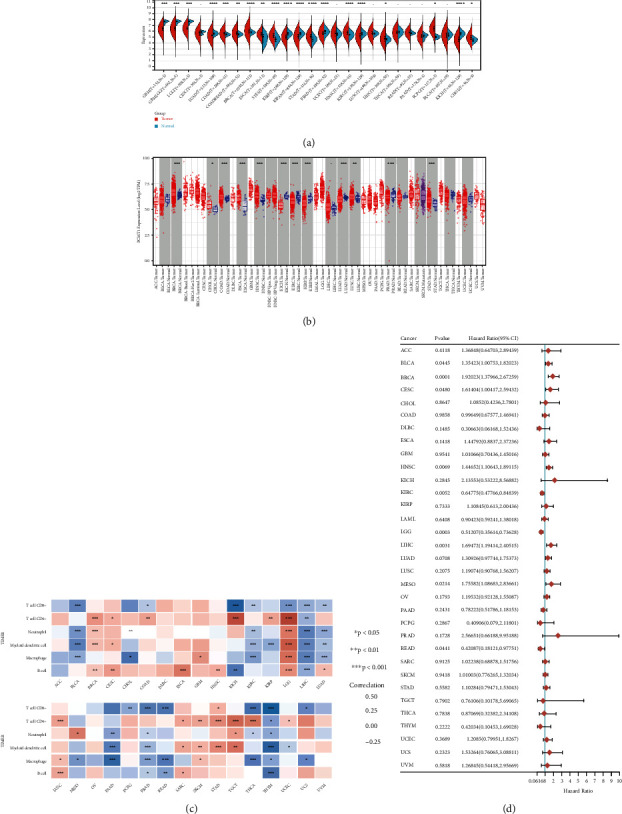
(a) Gene expression: the expression distribution of *PCMT1* gene in tumor tissues and normal tissues. (b) Differential expression of *PCMT1* in pancancer (TIMER). (c) Immune correlation: a heat map of the correlation between *PCMT1* gene expression and immunity in multiple tumor tissues. The significance of the two groups of samples passed the Wilcox test. (∗*P* < 0.05, ∗∗*P* < 0.01, ∗∗∗*P* < 0.001). (d) Forest plot: the correlation between *PCMT1* gene and prognosis in multiple tumors.

We used R software to calculate the expression difference between normal samples and tumor samples in each tumor and used unpaired Wilcoxon rank Sum and signed rank tests to analyze the significance of the difference. We observed that the *PCMT1* gene was significantly up-regulated in 10 tumors including colon adenocarcinoma (COAD, tumor: 5.57 ± 0.42; normal: 5.44 ± 0.19, *P* = 8.4e − 4) and breast carcinoma (BRCA, tumor: 6.04 ± 0.60; normal: 5.79 ± 0.20, *P* = 9.8e − 11) (Figure 1(a)). The TIMER database also yielded similar results (Figure 1(b)). Then, we used univariate Cox regression analysis to evaluate the prognosis of *PCMT1* for a variety of human cancers. The results are shown in Figure 1(d). *PCMT1* has a good prognostic effect on a variety of cancers, including BRCA (HR: 1.9 (1.3, 2.67), *P* = 0.001). The results of immune correlation evaluation showed that in addition to adrenocortical carcinoma (ACC), lymphoid neoplasm diffuse large B cell lymphoma (DLBC), and uveal melanoma (UVM), *PCMT1* has varying degrees of correlation with the immune infiltrating cells of a variety of human cancers (Figure 1(c)). We also evaluated the expression of immune checkpoint-related genes in different tumor tissues (see Supplementary Figure 1). The results showed that with the exception of ACC, cholangiocarcinoma (CHOL), DLBC, and esophageal carcinoma (ESCA), there was a significant correlation between the expression of most tumors and immune checkpoint-related genes. In addition, we also assessed the correlation between tumor mutation burden and *PCMT1* expression (See Supplementary Figure 2). The results show that there is a significant positive correlation between *PCMT1* expression and tumor mutation burden in multiple tumor types, including ACC, uterine carcinoma (UCS), and BRCA.

### 3.2. PCMT1 Is Overexpressed in Breast Cancer

**Figure 2:**
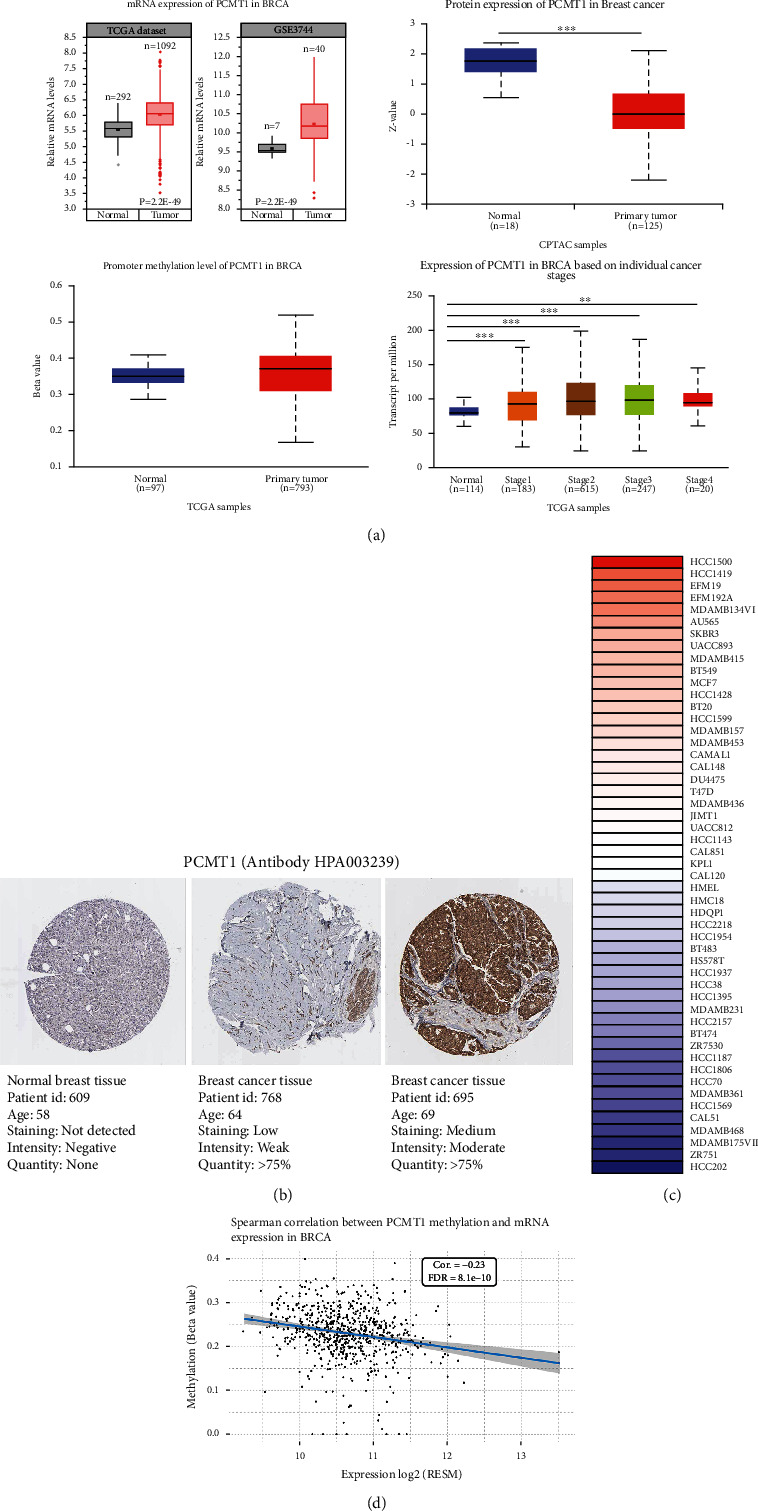
(a) The expression of *PCMT1* mRNA and protein in breast cancer, the level of *PCMT1* methylation, and the correlation with tumor staging. (b) The expression of *PCMT1* in normal tissues and breast cancer tissues in HPA database. (c) The expression of *PCMT1* in multiple breast cancer cell lines. (d) Correlation of PCMT1 mRNA expression and methylation in BRCA.

Based on the TCGA-BRCA data set, we obtained the differential expression of *PCMT1* in breast cancer tissues and normal tissues. The expression of *PCMT1* in breast cancer tissues was significantly higher than that in normal tissues (*P* = 2.2E − 49), and this differential expression was also verified in GSE3744 (*P* = 5.7E − 5) (Figure 2(a)). We used the UALCAN online database to compare the expression levels of PCMT1 protein in normal and breast tissues. The protein expression level of PCMT1 in breast cancer tissues (*n* = 125) was lower than that in normal tissues (*n* = 18) (Figure 2(a)). To account for this difference, we analyzed the correlation of *PCMT1* methylation and mRNA expression in breast cancer using the gene set cancer analysis (GSCA) database, which showed a significant negative correlation. Therefore, we speculate that the inconsistency of *PCMT1* mRNA and protein expression in breast cancer stems from the inhibition of posttranslational modification and other processes in breast cancer, resulting in lower protein expression levels than normal tissues. The results are shown in Figure 2(d). There was no significant difference in the methylation level of *PCMT1* in breast cancer (*n* = 793) and normal tissues (*n* = 97). In addition, we assessed the association between the expression of *PCMT1* in breast cancer and tumor staging. The results showed that the expression level of *PCMT1* was significantly correlated with the rising trend of breast cancer tumor stages (Figure 2(a)). Using the Human Protein Atlas database, we assessed the expression level of *PCMT1* through immunohistochemical images of *PCMT1* in clinical breast cancer samples. The results showed that *PCMT1* was overexpressed in tumor tissues (Figure 2(b)). Based on the CCLE database, we explored the expression level of *PCMT1* in different breast cancer cell lines, and the expression level of *PCMT1* was significantly different in different breast cancer cell lines. The heat map shows that *PCMT1* is highly expressed in human breast ductal carcinoma cell lines, such as HCC1500, HCC1419, and EFM19 cell lines, while the expression is lowest in the HCC202 cell line (human breast primary ductal carcinoma cells) (Figure 2(c)).

We used bc-GenExMiner v4.7 to explore the relationship between PCMT1 and clinical pathological parameters of breast cancer. We observed that PCMT1 has a higher expression level in the age group over 51 years old (*P* < 0.0001). PCMT1 expressed higher in the negative progesterone receptor state (*P* = 0.0042). Compared with the human epidermal growth factor receptor 2 (HER2) negative group, the expression level of PCMT1 in the HER2 positive group was higher (*P* = 0.0294). In addition, for P53 status, we also observed that the mutant group had higher PCMT1 expression (*P* = 0.0001) (Table 1).

### 3.3. Gene Mutation and Immune Infiltration Level of PCMT1 in Breast Cancer

**Figure 3:**
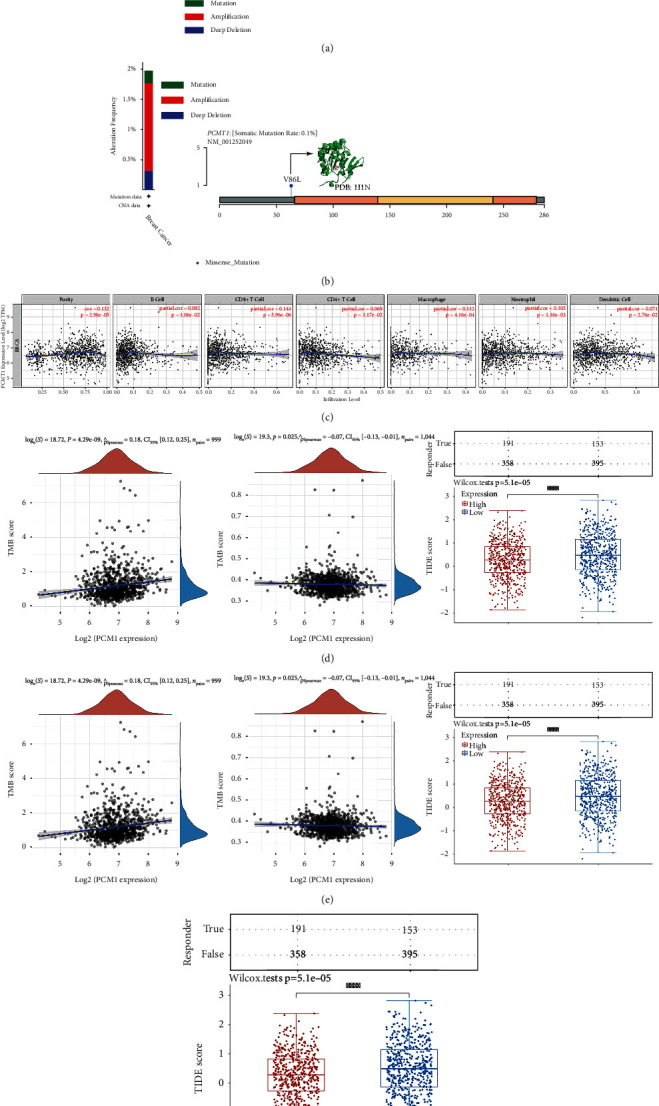
(a) Analysis of pancancer mutations of *PCMT1*. (b) Analysis of *PCMT1* mutations in breast cancer. (c) Correlation between *PCMT1* and the abundance of immune infiltration in breast cancer. (d) Correlation between *PCMT1* and TMB. (e) Correlation between *PCMT1* and MSI. (f) The relationship between *PCMT1* expression level and ICB response.

First, based on the existing ICGC/TCGA-Nature 2020 data set (2922 patients) [39] on the cBioPortal platform, we conducted a genome-wide pancancer analysis of *PCMT1* gene mutations. The results showed that *PCMT1* has the highest mutation frequency in soft tissue sarcoma, esophagogastric cancer, and breast cancer (Figure 3(a)). Then, we studied the *PCMT1* gene mutation in breast cancer patients of TCGA Firehose Legacy (*n* = 1108 patients) [40]. Among the 1108 patients inquired, 19 (2%) patients had mutations in the *PCMT1* gene, and the main types of mutations included missense mutations, increased gene duplication, and deletion mutations. The 86th amino acid in PCMT1 is prone to mutation, and the mutation type is a missense mutation (Figure 3(b)).

**Figure 4:**
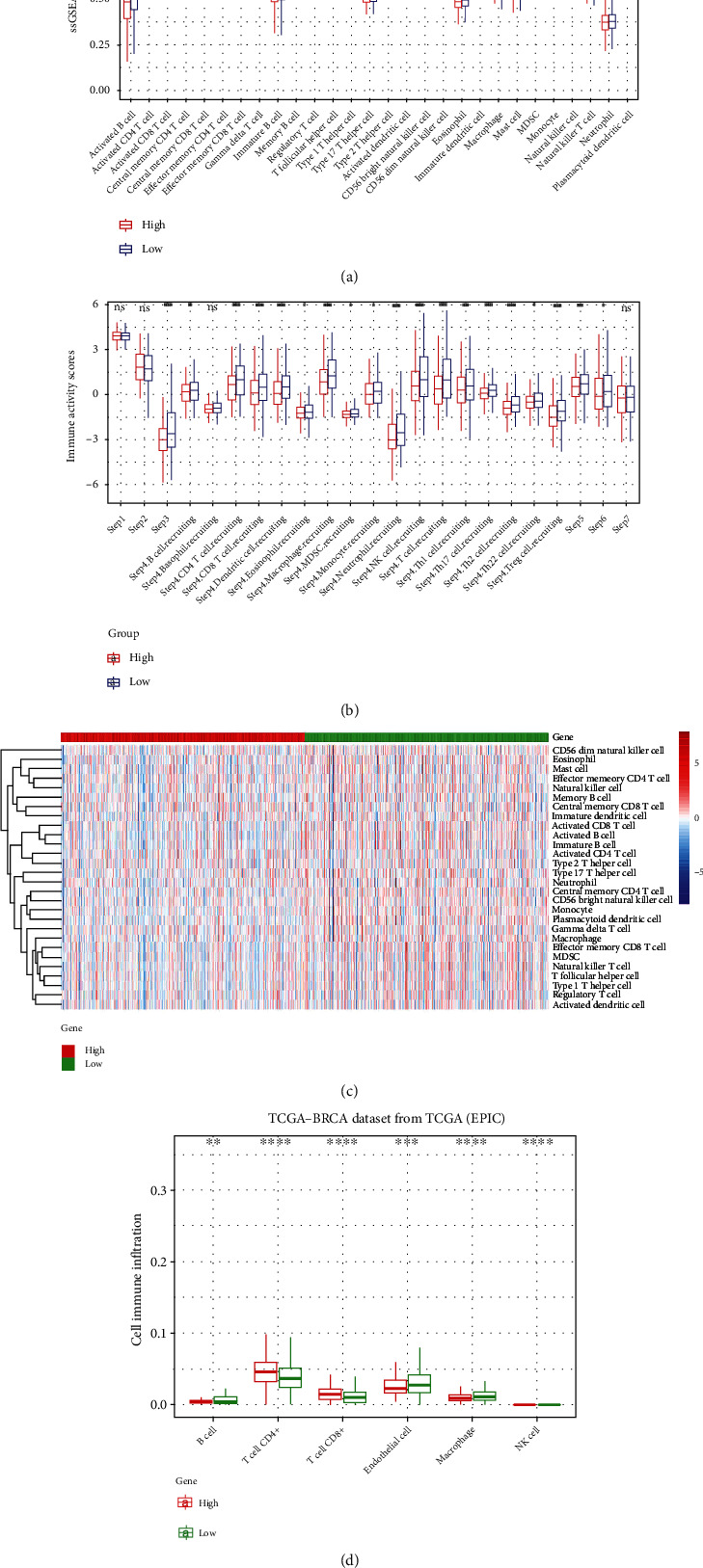
(a) Correlation between *PCMT1* expression and immune cell infiltration in breast cancer (ssGSEA, boxplot). (b) Correlation between *PCMT1* expression and seven-step anticancer immune status in breast cancer (TIP database). (c) Correlation between *PCMT1* expression and immune cell infiltration in breast cancer (ssGSEA, heatplot). (d) Correlation between *PCMT1* expression and immune cell infiltration in breast cancer (EPIC).

In addition, using the online server TIMER, we evaluated the relationship between the expression of *PCMT1* and tumor immune infiltrating cell biomarkers, including B cells, CD8+ T cells, CD4+ T cells, macrophages, neutrophils, and dendritic cells. The results showed that *PCMT1* was significantly positively correlated with tumor purity (*r* = 0.132, *P* = 2.96e − 05), B cells (*r* = 0.082, *P* = 1.06e − 02), CD8+ T cells (*r* = 0.144, *P* = 5.96e − 06), macrophages (*r* = 0.112, *P* = 4.16e − 04), neutrophils (*r* = 0.105, *P* = 1.16e − 03), and dendritic cells (*r* = 0.071, *P* = 2.76e − 02) and significantly negatively correlated with CD4+ T cells (*r* = −0.069, *P* = 3.17e − 02) (Figure 3(c)). EPIC analysis showed that the expression of *PCMT1* was significantly correlated with B cell, T cell CD4+, T cell CD8+, endothelial cell, and macrophage and NK cell (Figure 4(d)), and ssGSEA analysis also showed that *PCMT1* expression was associated with various immune cell infiltration (Figures 4(a) and 4(c)). TIP analysis showed that *PCMT1* was significantly associated with breast cancer priming and activation (step 3), trafficking of immune cells to tumors (step 4), infiltration of immune cells into tumors (step 5), and recognition of cancer cells by T cells (step 6) (Figure 4(b)). These results support the involvement of *PCMT1* in immune cell infiltration in breast cancer. *PCMT1* has a significant positive correlation with TMB (*r* = 0.18, *P* = 4.29e − 09) (Figure 3(d)), and a significant negative correlation with MSI (*r* = −0.07, *P* = 0.025) (Figure 3(e)). We also evaluated the relationship between *PCMT1* expression level and ICB response based on the TIDE algorithm. The results showed that high *PCMT1* expression has a lower TIDE score, ICB curative effect is better, and survival after ICB treatment is longer (Figure 3(f)).

### 3.4. Analysis of PCMT1 Coexpressed Genes in Breast Cancer

**Figure 5:**
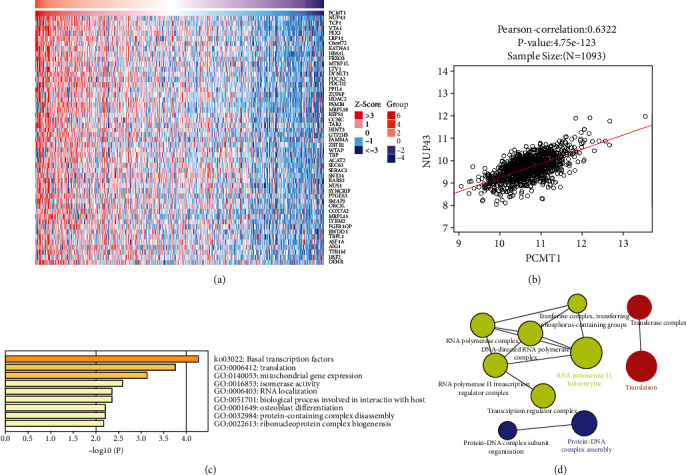
(a) Significantly positively correlated genes with *PCMT1* in breast cancer. (b) The correlation between *PCMT1* and *NUP43* expression in breast cancer by the LinkedOmics dataset. (c) Functional enrichment analysis of *PCMT1* co-expressed genes. (d) Network diagram of the first 50 co-expressed genes of *PCMT1*. Only pathways with *P* < 0.05 are shown, with the statistical option as a two-sided hypergeometric test for enrichment and Bonferroni for *P* value correction.

To further explore the potential mechanism of *PCMT1* in breast cancer, we used the LinkedOmics database to study the coexpression of *PCMT1*. The results showed that there was a significant positive correlation between *PCMT1* and *NUP43* (*r* = 0.6322, *P* = 4.75*e* − 123) (Figures 5(a) and 5(b)). Studies have shown that the upregulation of *NUP43* is associated with poorer OS in luminal A and HER2+ breast tumors [41]. Functional enrichment analysis was performed on the first 50 coexpressed genes, including biological process (BP), cellular component (CC), molecular function (MF), and Kyoto Encyclopedia of Genes and Genomes (KEGG). BP analysis showed that *PCMT1*-related coexpressed genes were mainly enriched in DNA-templated transcription, initiation, and translation. CC analysis showed that *PCMT1*-related coexpressed genes were mainly enriched in RNA polymerase II, holoenzymes, and mitochondrial matrix; MF analysis showed that *PCMT1*-related coexpressed genes were mainly enriched in isomerase activity; and KEGG analysis showed that *PCMT1*-related coexpressed genes were mainly enriched in basal transcription factors (Figures 5(c) and 5(d)).

### 3.5. Survival Analysis of PCMT1

**Figure 6:**
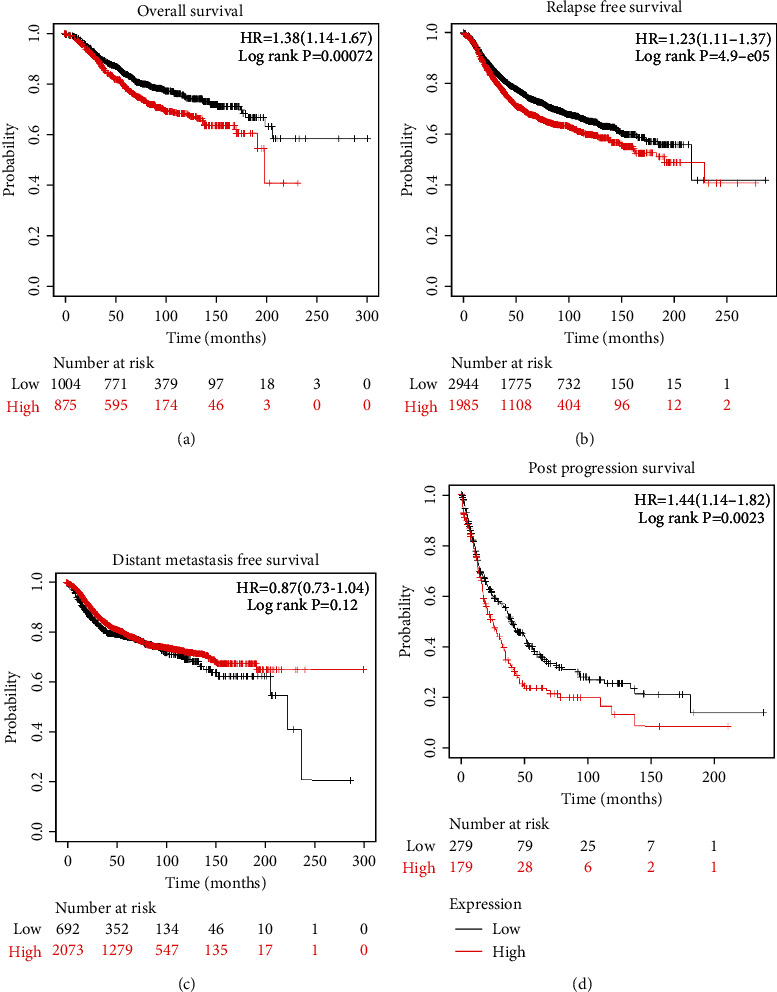
Survival analysis of *PCMT1* in breast cancer, including OS, RFS, DMFS, and PPS.

Based on the online server Kaplan–Meier plotter, we evaluated the relationship between the expression level of *PCMT1* and the survival of breast cancer patients to reveal the prognostic value of *PCMT1* in breast cancer. The results showed that low expression of *PCMT1* was significantly correlated with longer OS (*HR* = 1.38, 95% CI: 1.14–1.67, *P* = 0.00072), RFS (*HR* = 1.23, 95% CI: 1.11–1.37, *P* = 4.9e − 05), and PPS (*HR* = 1.44, 95% CI: 1.14–1.82, *P* = 0.0023) in breast cancer patients, but not significantly correlated with DMFS (*HR* = 0.87, 95% CI: 0.73–1.04, *P* = 0.12) (Figure 6). This suggests that *PCMT1* may have the function of a biomarker for the early diagnosis of breast cancer.

In addition, we analyzed the prognostic value of PCMT1 mRNA expression in different molecular subtypes according to the 2013 St. Gallen breast cancer criteria, including basal-like, luminal A, luminal B, and HER2+ subtypes. We evaluated the three probes (205202_at, 208857_s_at, 210156_s_at) in the Kaplan–Meier Plotter database. Survival outcomes include RFS, OS, DMFS, and PPS. We observed that high expression of PCMT1 was significantly associated with poor prognosis in the luminal A subtype (Table 2). The high expression of PCMT1 is significantly correlated with the worse RFS, OS, DMFS, and PPS of luminal A subtype.

### 3.6. The Relationship between the PCMT1 Gene and the Prognosis of Breast Cancer

**Figure 7:**
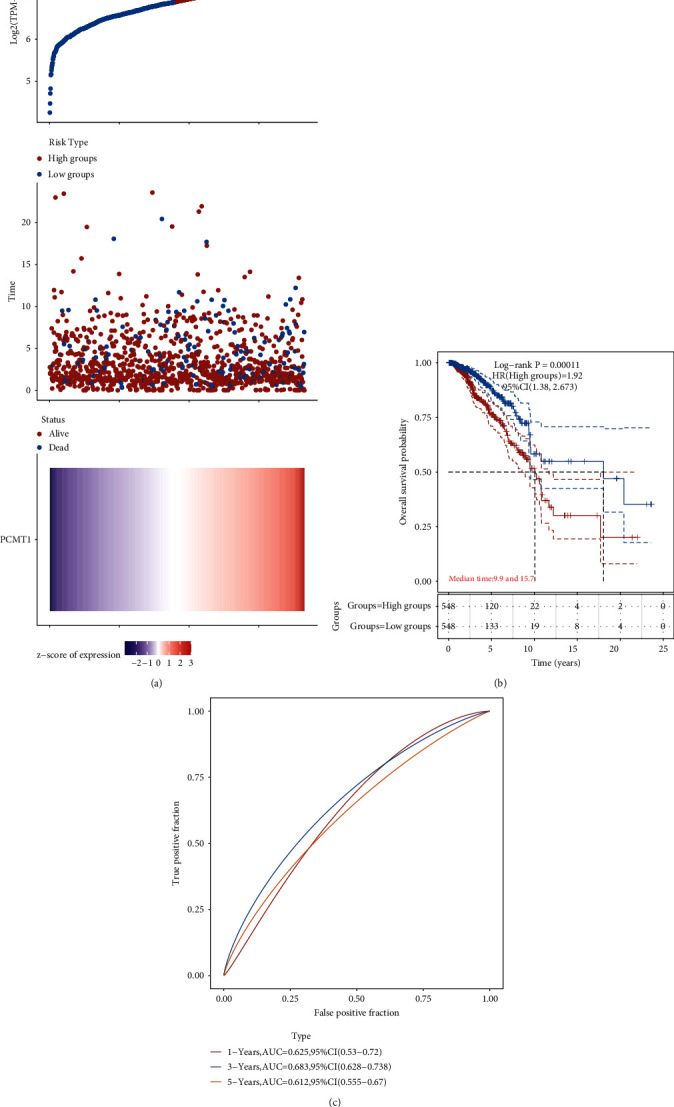
(a) The relationship between *PCMT1* gene expression (top figure) and survival time (middle figure), and survival status (bottom figure) in the TCGA data set. The abscissas of the three figures all represent samples, and the order of the samples is consistent. (b) KM survival curve distribution of the gene in the TCGA data set. (c) The ROC curve and AUC of the gene at different times, where the higher the AUC value, the stronger the predictive ability of the gene.

Based on the raw count of the RNA sequencing data of 1097 breast cancer tumors downloaded from the TCGA data set and the corresponding clinical information, we studied the prognostic effect of the *PCMT1* gene on breast cancer. We arranged the samples in order of *PCMT1* expression level from high to bottom and used different grouping methods to analyze the prognostic differences of different groups. The results showed that *PCMT1* gene expression and survival time in the TCGA data set were positively correlated with survival status (Figure 7(a)). The KM survival curve was drawn based on the TCGA data set and showed that high *PCMT1* expression may be a risk factor for poor prognosis in breast cancer patients (HR: 1.92 (1.38, 2.67), *P* = 0.0001) (Figure 7(b)). AUC can observe the effectiveness of *PCMT1* as a prognostic biomarker. We drew the 1-year, 3-year, and 5-year ROC curves of the *PCMT1* gene and calculated the AUC value. Among them, at 3 years, the AUC value was 0.683, indicating that *PCMT1* has a certain diagnostic value.

Given that NUP43 is significantly associated with the prognosis of different subtypes of breast cancer, we analyzed the prognosis of *PCMT1* in different subtypes of breast cancer, and the results showed that high expression of *PCMT1* was significantly associated with poor prognosis in the luminal A subtype (see Supplementary Figure 3).

### 3.7. Construction and Verification of Nomogram Based on PCMT1

First, univariate Cox regression analysis was used to show that the expression of *PCMT1* (HR: 2.245; CI: 1.54–3.28; *P* = 3e − 05), PT staging (HR: 1.70; CI: 1.30–2.20; *P* = 7e − 05), and PN staging (HR: 1.82; CI: 1.47–2.26; *P* < 0.0001) was significantly related to prognosis. Multivariate Cox regression analysis showed that the expression of *PCMT1* (HR: 2.46; CI: 1.62–3.73; *P* = 0.00002), PT staging (HR: 1.48; CI: 1.10–1.99; *P* = 0.009), and PN staging (HR: 1.70; CI: 1.34–2.16; *P* = 0.00001) was also significant, indicating that the *PCMT1* gene is a variable independent of other clinical factors.

**Figure 8:**
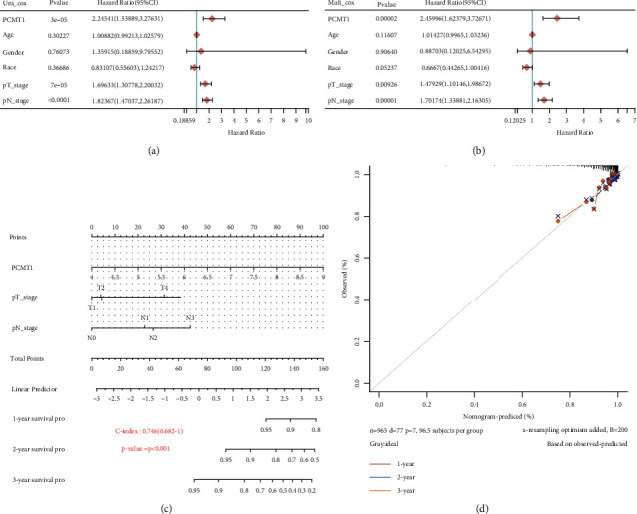
(a) Univariate Cox analysis of *PCMT1* gene expression and clinical characteristics. (b) Multivariate Cox analysis of *PCMT1* gene expression and clinical characteristics. (c) The nomogram can predict the overall survival of breast cancer patients at 1, 2, and 3 years. (d) Calibration curve for the disease specific survival nomogram model.

We constructed nomograms using *PCMT1* and independent clinical risk factors to provide a quantitative method for predicting disease-specific survival outcomes in breast cancer patients and luminal A subtypes. The 45° line represents the best prediction. In addition, we also analyzed the prediction efficiency of the nomogram, and the results showed that the C-index of the model was 0.746 (CI: 0.682-1) and 0.757 (CI: 0.689-1), respectively. It is shown that the model has good prediction accuracy for breast cancer prognosis (especially luminal A) (see Figure 8 and Supplementary Figure 4).

## 4. Discussion

Initial research showed the antiapoptotic effect of *PCMT1* and speculated that its main mechanism is the ability to maintain the structural stability of some key antiapoptotic proteins through methylation and repair of some malfunctioning proteins [42]. The role of PCMT1 was subsequently proven in liver cancer. Studies have shown that *PCMT1* is effectively regulated by the microRNA 15a/16-1 cluster and participates in cell apoptosis by protecting the structural stability and biological functions of BclxL (antiapoptotic mediator) from deamidation [43]. This proves that *PCMT1* is involved in the regulation of hepatoma cell apoptosis. Zhao et al. constructed a random tumor transcriptome expression library to successfully create an A5 protein antigen targeting *PCMT1* and showed a significant immunotherapy effect on S180 sarcoma [44]. Saito et al. proved that *PCMT1* overexpression is an independent predictor of poor prognosis of lung adenocarcinoma through multivariate Cox risk regression analysis [45]. In addition, a study of *PCMT1* in bladder cancer showed that *PCMT1* regulates the migration and invasion of bladder cancer cells, promotes the occurrence and development of bladder cancer, and emphasizes that *PCMT1* is an unfavorable prognostic biomarker for bladder cancer [15].

Although it has been confirmed that *PCMT1* is involved in the occurrence and development of several cancers and its prognostic role has been emphasized, the role of *PCMT1* in breast cancer has not been confirmed. In our study, we first performed a pancancer analysis of *PCMT1*, and the results showed that *PCMT1* is highly expressed in a variety of cancers, including confirmed lung adenocarcinoma. The results of pancancer immune correlation evaluation show that, in addition to ACC, DLBC, and UVM, *PCMT1* has varying degrees of correlation with the immune infiltrating cells of a variety of human cancers. Pancancer analysis also revealed that the expression of *PCMT1* is significantly correlated with the prognosis of a variety of cancers. TMB is a biomarker that can help predict the patient's response to immunotherapy [46]. We evaluated the correlation between TMB and *PCMT1* expression in a variety of cancers, and the results showed that the expression of *PCMT1* was significantly correlated with a variety of cancers, including ACC and BRCA. This finding suggests that *PCMT1* may participate in the regulation of a variety of tumor-related signaling pathways and is significantly related to immune infiltration. Then, we specifically studied the expression of *PCMT1* in breast cancer. Compared with normal tissues, the *PCMT1* gene is significantly more highly expressed in breast cancer, but its protein level is lower than that in normal tissues, revealing the influence of posttranslational modifications in breast cancer on *PCMT1*. In addition, the expression of *PCMT1* is significantly related to the tumor stage of breast cancer, suggesting that it may be a prognostic marker of breast cancer. The analysis of breast cancer immune infiltration indicated that *PCMT1* was significantly related to biomarkers of breast cancer immune infiltrating cells. The survival analysis of *PCMT1* in breast cancer showed that the high expression of *PCMT1* can lead to shorter OS, RFS, and PPS in breast cancer patients, suggesting that high expression of *PCMT1* is significantly related to the poor prognosis of breast cancer. In addition, we constructed an ROC curve and a nomogram to observe the efficacy of *PCMT1* as a prognostic biomarker. The results showed that the *PCMT1* gene is a variable independent of other clinical factors and can guide the prognosis of breast cancer.

The limitation of our research is mainly reflected in the fact that all researches are based on the results of bioinformatics analysis. The potential biological mechanism of *PCMT1* in breast cancer, its potential relationship with tumor immune escape, and its clinical role still need to be further studied. Our research results will provide certain reference value for further research on the role of *PCMT1* in breast cancer.

## 5. Conclusion

In summary, our results show that the high expression of *PCMT1* is significantly related to the poor prognosis of breast cancer, may be a potential biomarker of breast cancer, and is significantly related to the immune infiltration of breast cancer.

## Figures and Tables

**Table 1:** The relationship between PCMT1 and the clinicopathological parameters of breast cancer (bc-GenExMiner v4.7).

Clinical parameters	PCMT1	
	No.	*P* value
Age		
≤ 51	2947	**<0.0001**
> 51	4796∗	
Nodal status		
Negative	4408	0.1226
Positive	3717	
ER (IHC)		
Negative	2606	0.6540
Positive	7210	
PR (IHC)		
Negative	2727∗	**0.0042**
Positive	3483	
HER2(IHC)		
Negative	4768	**0.0294**
Positive	794∗	
Triple-negative status		
Not	7744	0.2142
TNBC	940	
P53 status (IHC)		
Wild type	638	**0.0001**
Mutated	284∗	

All the data of the PCMT1 were based on bc-GenExMiner v4.7. “No.” means the patients' number from database; “∗” means higher mRNA expression level compared with the same group. ER: estrogen receptor; PR: progesterone receptor; HER2: human epidermal growth factor receptor 2; TNBC: triple-negative breast cancer. The data with statistical significance (*P* < 0.05) were marked in bold text.

**Table 2:** Survival analyses of PCMT1 with different molecular subtypes based on the 2013 St. Gallen criteria in breast cancer.

BRCA subtype	Survival outcome	205202_at		208857_s_at		210156_s_at	
		HR (95% CI)	*P*	HR (95% CI)	*P*	HR (95% CI)	*P*
Basal-like	RFS	1.12 (0.89-1.4)	0.34	0.9 (0.71-1.14)	0.38	0.92 (0.74-1.15)	0.47
	OS	1.3 (0.84-2)	0.23	0.63 (0.43-0.94)	**0.022**	0.6 (0.41-0.88)	**0.0084**
	DMFS	0.73 (0.52-1.03)	0.071	0.69 (0.5-0.94)	**0.018**	0.86 (0.62-1.21)	0.39
	PPS	1.39 (0.73-2.64)	0.31	0.55 (0.31-0.95)	**0.029**	0.72 (0.41-1.28)	0.26
Luminal A	RFS	1.43 (1.21-1.69)	**2.7e-05**	1.64 (1.38-1.95)	**1.1e-08**	1.23 (1.04-1.47)	**0.019**
	OS	1.94 (1.41-2.67)	**3.9e-05**	2.04 (1.48-2.81)	**8.5e-06**	1.34 (0.97-1.85)	0.075
	DMFS	1.32 (1.02-1.71)	**0.036**	1.63 (1.24-2.15)	**0.00045**	1.34 (1.02-1.76)	**0.038**
	PPS	1.78 (1.24-2.55)	**0.0014**	2.24 (1.55-3.24)	**1.2e-05**	0.75 (0.51-1.09)	0.13
Luminal B	RFS	1.4 (1.13-1.72)	**0.0017**	1.52 (1.28-1.82)	**2.5e-06**	1.37 (1.15-1.64)	**0.0004**
	OS	1.42 (0.93-2.19)	0.1	1.51 (1.06-2.14)	**0.02**	1.27 (0.85-1.9)	0.24
	DMFS	0.83 (0.61-1.15)	0.26	0.82 (0.62-1.1)	0.18	1.19 (0.88-1.6)	0.26
	PPS	1.7 (0.99-2.93)	0.053	1.3 (0.85-2.27)	0.19	0.86 (0.56-1.31)	0.49
HER2+	RFS	1.31 (0.88-1.96)	0.19	0.67 (0.44-1.02)	**0.063**	0.72 (0.5-1.04)	0.075
	OS	0.8 (0.44-1.45)	0.47	0.51 (0.25-1.06)	**0.066**	0.51 (0.26-0.99)	**0.044**
	DMFS	0.48 (0.24-0.97)	**0.037**	0.48 (0.24-0.89)	**0.019**	0.59 (0.35-1.02)	0.055
	PPS	1.51 (0.57-3.98)	0.4	0.58 (0.25-1.38)	0.21	0.57 (0.24-1.35)	0.19

HR: hazard ratio; CI: confidence interval; RFS: relapse free survival; OS: overall survival; DMFS: distant metastasis-free survival; PPS: postprogression survival. All of the data above were obtained from the Kaplan–Meier Plotter database. The data with statistical significance (*P* < 0.05) were marked in bold text.

## Data Availability

All data generated or analyzed during the current study are included in this published article and its supplementary information files.

## Supplementary Materials

Supplementary Materials Supplementary Figure 1 The expression of immune checkpoint-related genes in different tumor tissues. Supplementary Figure 2 The correlation between tumor mutation burden and *PCMT1* expression. Supplementary Figure 3 Relationship between *PCMT1* and prognosis of different BRCA subtypes. Supplementary Figure 4 Construction of a predictive model of *PCMT1* for luminal A isoforms.
