# Supplementary material for: PCMT1 Is a Potential Prognostic Biomarker and Is Correlated with Immune Infiltrates in Breast Cancer
Source: Biomed Res Int. 2022 Apr 30;2022:4434887. doi: 10.1155/2022/4434887 (PMC9078795; doi:10.1155/2022/4434887)

**Supplementary Figure 1 The expression of immune checkpoint-related genes in different tumor tissues**


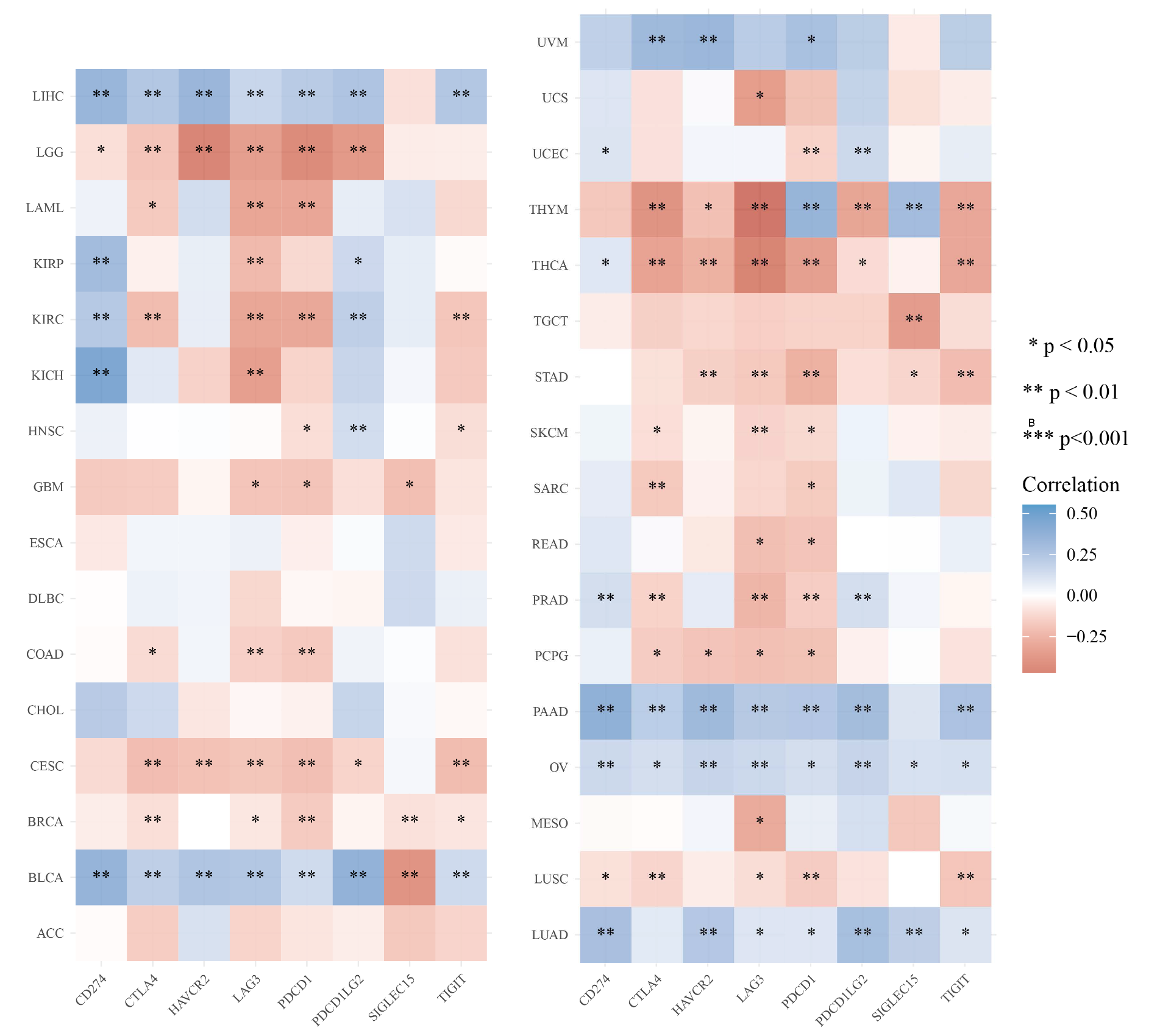


**Supplementary Figure 2 The correlation between tumor mutation burden and *PCMT1* expression**


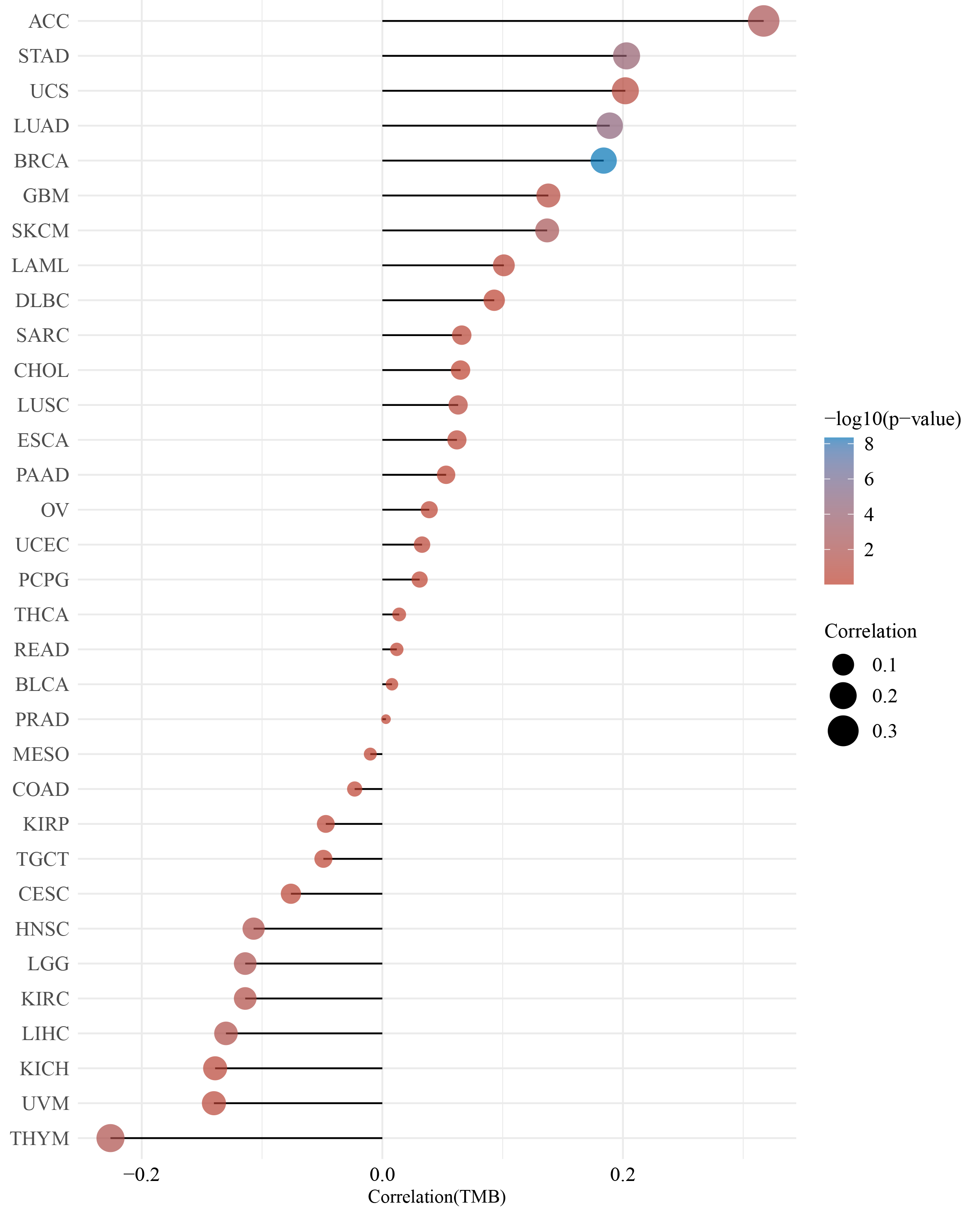


**Supplementary Figure 3 Relationship between PCMT1 and prognosis of different BRCA subtypes**


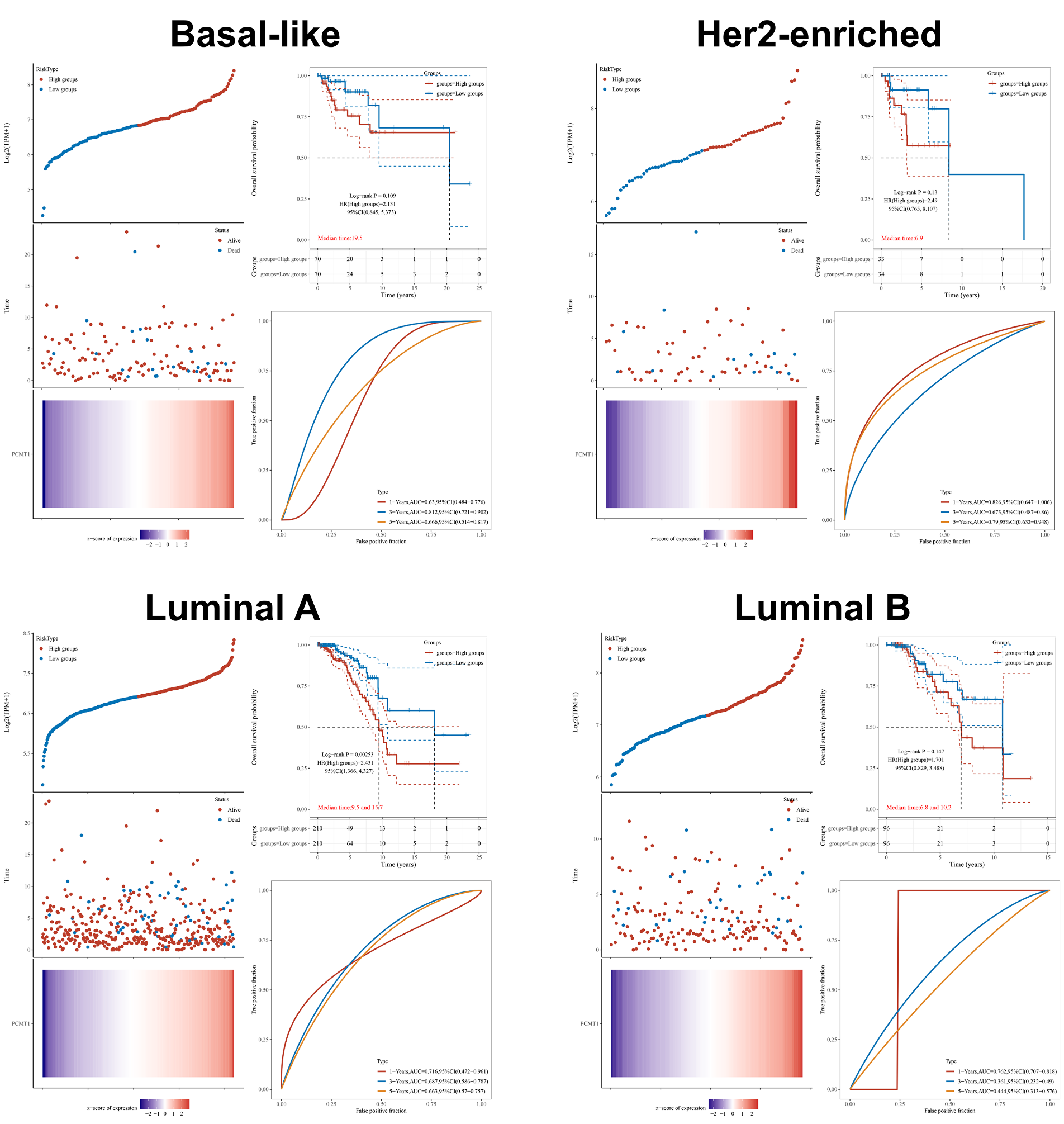


**Supplementary Figure 4 Construction of a predictive model of PCMT1 for luminal A isoforms**


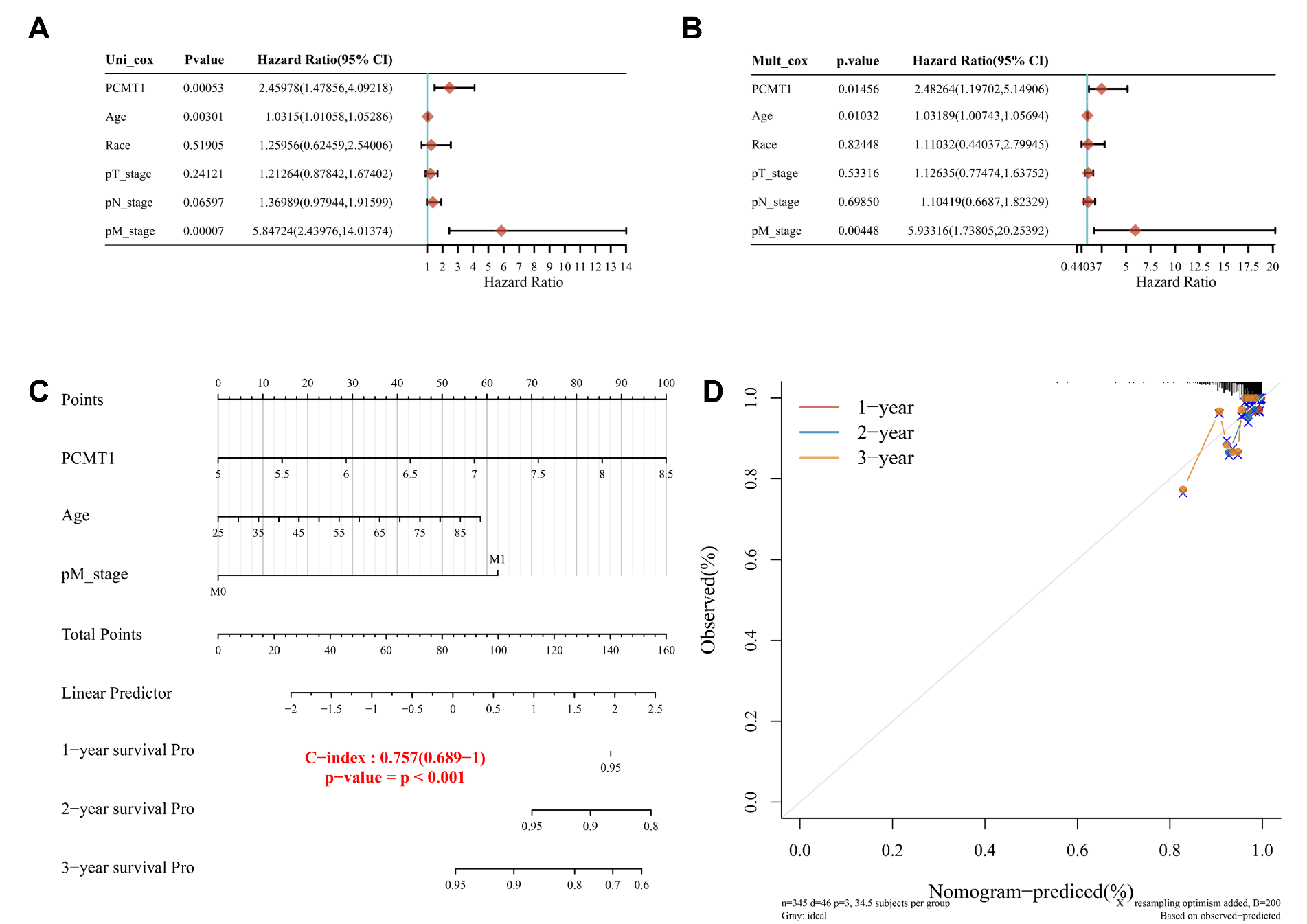

Supplement: Supplementary Materials — Supplementary Figure 1 The expression of immune checkpoint-related genes in different tumor tissues. Supplementary Figure 2 The correlation between tumor mutation burden and PCMT1 expression. Supplementary Figure 3 Relationship between PCMT1 and prognosis of different BRCA subtypes. Supplementary Figure 4 Construction of a predictive model of PCMT1 for luminal A isoforms. [file 4434887.f1.docx]
